# Comparative assessment of the bacterial communities associated with *Aedes aegypti* larvae and water from domestic water storage containers

**DOI:** 10.1186/1756-3305-7-391

**Published:** 2014-08-24

**Authors:** Nsa Dada, Estelle Jumas-Bilak, Sylvie Manguin, Razak Seidu, Thor-Axel Stenström, Hans J Overgaard

**Affiliations:** Department of Mathematical Sciences and Technology, Norwegian University of Life Sciences, Ås, Norway; Laboratoire d’Hygiène hospitalière, Centre Hospitalier Régional Universitaire de Montpellier, Montpellier, France; UMR5119, Université Montpellier 1, Montpellier, France; IRD2 Institut de Recherche pour le Développement (IRD), UMR-MD3, Université Montpellier 1, Montpellier, France; SARChl Chair, Institute for Water and Waste Water Technology, Durban University of Technology, Durban, South Africa; Institut de Recherche pour le Développement (IRD), Maladies Infectieuses et Vecteurs, Ecologie, Génétique, Evolution et Contrôle (IRD 224-CNRS 5290 UM1-UM2), Montpellier Cedex 5, France; Department of Entomology, Faculty of Agriculture, Kasetsart University, Bangkok, 10900 Thailand

**Keywords:** *Aedes aegypti*, Bacterial diversity, Enteric bacteria, *E. coli*, Domestic water storage containers, 16S rRNA-TTGE, Thailand

## Abstract

**Background:**

Domestic water storage containers constitute major *Aedes aegypti* breeding sites. We present for the first time a comparative analysis of the bacterial communities associated with *Ae. aegypti* larvae and water from domestic water containers.

**Methods:**

The 16S rRNA-temporal temperature gradient gel electrophoresis (TTGE) was used to identify and compare bacterial communities in fourth-instar *Ae. aegypti* larvae and water from larvae positive and negative domestic containers in a rural village in northeastern Thailand. Water samples were cultured for enteric bacteria in addition to TTGE. Sequences obtained from TTGE and bacterial cultures were clustered into operational taxonomic units (OTUs) for analyses.

**Results:**

Significantly lower OTU abundance was found in fourth-instar *Ae. aegypti* larvae compared to mosquito positive water samples. There was no significant difference in OTU abundance between larvae and mosquito negative water samples or between mosquito positive and negative water samples. Larval samples had significantly different OTU diversity compared to mosquito positive and negative water samples, with no significant difference between mosquito positive and negative water samples. The TTGE identified 24 bacterial taxa, belonging to the phyla Proteobacteria, Firmicutes, Actinobacteria, Bacteroidetes and TM7 (candidate phylum). Seven of these taxa were identified in larval samples, 16 in mosquito positive and 13 in mosquito negative water samples. Only two taxa, belonging to the phyla Firmicutes and Actinobacteria, were common to both larvae and water samples. Bacilli was the most abundant bacterial class identified from *Ae. aegypti* larvae, Gammaproteobacteria from mosquito positive water samples, and Flavobacteria from mosquito negative water samples. Enteric bacteria belonging to the class Gammaproteobacteria were sparsely represented in TTGE, but were isolated from both mosquito positive and negative water samples by selective culture.

**Conclusions:**

Few bacteria from water samples were identified in fourth-instar *Ae. aegypti* larvae, suggesting that established larval bacteria, most likely acquired at earlier stages of development, control the larval microbiota. Further studies at all larval stages are needed to fully understand the dynamics involved. Isolation of enteric bacteria from water samples supports earlier outcomes of *E. coli* contamination in *Ae. aegypti* infested domestic containers, suggesting the need to further explore the role of enteric bacteria in *Ae. aegypti* infestation.

## Background

The container-breeding mosquito, *Aedes aegypti*, is a well-recognized vector of diseases of significant public health concern, such as dengue fever, yellow fever, and chikungunya [1]. *Aedes aegypti* is known to breed mainly in domestic water containers in and around human dwellings [2–4]. These containers are ecosystem microcosms supporting food webs that are dependent on bacteria [5]. Studies conducted on the microbial ecology of *Aedes* breeding containers have mainly focused on the invasive *Ae. albopictus* as well as other tree-hole mosquitoes e.g. *Ae. triseriatus* and *Toxorhynchites rutilus*. These studies have explored the microbial communities in natural containers such as tree holes, discarded or unused containers (e.g. tyres), and ornamental containers (e.g. plant pots and cemetery urns) [5–8] with no focus on the microbial ecology of *Ae. aegypti* infested domestic water containers. One study on the effect of *Ae. aegypti* midgut microbiota on its susceptibility to DENV-2 however characterized the bacterial content of domestic water containers from which the mosquitoes were collected [9].

Mosquito-microbe interactions are of increasing research interests [10]. These interactions which span from pathogenic to obligate symbiosis [11], usually affect the evolutionary success and physiological functions of the mosquitoes. Such functions could be beneficial to the mosquitoes, for example, in the synthesis of essential nutrients that may be lacking from food sources, and conferral of resistance to pathogens [10, 12], or detrimental, where the microbiota directly interfere with mosquito development and fitness [10, 13]. These interactions are being explored for the development of novel control strategies for mosquito vectors and mosquito-borne diseases [12], an approach recently termed ‘symbiotic control’ [14].

Bacteria constitute a proportion of mosquito microbiota, colonizing a variety of mosquito organs, chiefly the midgut, and to a lesser extent the hemolymph, salivary glands and reproductive organs [10, 11]. The bacterial communities within mosquitoes vary depending on the mosquito species, sex, stage of development and habitat [10]. The origin of bacteria within mosquitoes and the dynamics involved in bacterial colonization are unclear as both exogenous and endogenous factors are known to affect the initial colonization and nature of the bacterial composition [10]. Some studies show that immature mosquitoes or newly emerged adults harbor bacteria derived from their breeding habitats [10, 15]. Others show that the bacterial communities in blood feeding adults are influenced by their blood meals [16]. Feeding may, thus, play a role in determining bacterial communities within mosquitoes. The bacteria within mosquitoes have also been shown to be acquired transstadially [17]. Yet it is still uncertain whether these bacteria acquired transstadially or through feeding are able to survive and colonize the mosquitoes, or are transient and lost during digestion and/or molting [10]. A better understanding of the dynamics involved in bacterial colonization within mosquitoes is therefore still needed.

Furthermore, understanding the mosquito-bacteria interactions within *Ae. aegypti* infested domestic containers would be beneficial for *Ae. aegypti* control. Thus far, one study that has examined the bacterial content of *Ae. aegypti* infested domestic containers [9] employed conventional culture-dependent methods. These methods are unable to capture non-cultivable bacteria that may be present within samples, thus leading to an incomplete picture of the bacterial communities. This contributes to the vague knowledge or limited information available on the dynamics involved in the mosquito-bacteria interactions. However, molecular methods such as the temporal temperature gradient gel electrophoresis (TTGE) and 16S rRNA sequencing can identify both cultivable and non-cultivable bacteria although not without limitations [18].

In this study, the 16S rRNA-TTGE technique was used to comparatively assess the bacterial communities associated with *Ae. aegypti* larvae and water from domestic water containers. We hypothesized that the bacteria in *Ae. aegypti* infested containers are determinants of *Ae. aegypti* production and thus may constitute a major proportion of the larval microbiota. This was based on our previous study where significantly more *Ae. aegypti* pupae were produced in domestic containers contaminated with *Escherichia coli* compared to containers that were not contaminated with *E. coli* [19]. To test this hypothesis and update information on the bacterial communities associated with *Ae. aegypti* larvae and water in *Ae. aegypti* infested domestic containers, the abundance and diversity of bacterial taxa between *Ae. aegypti* larvae and water from *Ae. aegypti* positive and negative containers were determined and compared.

## Methods

### Sampling sites and sample collection

The study was conducted in February 2012 following our earlier study on the relationship between *Ae. aegypti* production and *E. coli* contamination in domestic containers in Thailand and Laos [19]. Mosquito and water samples were collected from domestic water containers in Waileum village, Khon Kaen province, Thailand, where 25 out of 122 houses included in the previous study were randomly selected. Out of the randomly selected houses, 17 had at least one mosquito positive container, and were included in this study. The other eight houses were excluded because they were either unoccupied at the time of sampling or had no mosquito positive containers.

In each house, water samples were collected from each mosquito positive container and one mosquito negative container directly into 100 ml standard Whirl-Pak® Thio-Bags®. Water was vigorously mixed prior to sampling to ensure that biofilms were included. A total of 25 *Ae. aegypti* positive containers (10 cement tanks, 12 earthen jars, two plastic buckets and one plastic drum), and 17 negative containers (13 earthen jars, two cement tanks, one plastic drum and one metal pot) were sampled. A sample of ten 4th instar larvae (or all if less than ten) was collected from each mosquito positive container and transferred to sterile 15 ml Eppendorf tubes. All water and larval samples were transported on dry ice to the laboratory and stored in -80°C until further processing.

### Sample preparation for DNA extraction

Samples were thawed at room temperature before DNA extraction. *Aedes aegypti* larvae were identified using illustrated keys [20], and all *Ae. aegypti* larvae (mean 4 ± 1) per container were pooled together for analysis. A total of 25 pools of *Ae. aegypti* larvae from 25 mosquito positive containers were analyzed. Larval samples were surface sterilized; first rinsed in 70% ethanol, then suspended in 70% ethanol and agitated with a vortex mixer for about 10 seconds, and finally rinsed with sterile DNA free water.

Each water sample was filtered through a cellulose nitrate membrane filter (0.45 μm pore size, 47 mm dia., Sartorius Stedium®) using aseptic vacuum filter units (Millipore®). 0.45 μm pore size membrane filters were used due to high turbidity of some water samples. Each membrane filter was cut in two halves; one half cut into small pieces and placed in sterile 1.5 ml Eppendorf tubes for DNA extraction and the other placed on Trypticase soy agar (TSA) plates for bacterial cultures and further isolation of enteric bacteria. Prior to water sample filtration, sterile DNA free water was filtered through each filter unit (using separate membranes) as negative controls to check for contamination.

### Isolation of enteric bacteria from water samples

One half of each filter membrane placed on TSA plates were incubated at 37 ± 0.5°C for 48 hours. Colonies from positive TSA plates were cryopreserved in Tryptic soy broth (TSB) with 15% glycerol at -80°C for isolation of enteric bacteria. Enteric bacteria were isolated from preserved samples by streaking on Drigalski agar (DA) plates. Following incubation at 37 ± 0.5°C for 48 hours, each colony displaying distinct morphotypes on positive DA plates were isolated and sub-cultured on fresh DA plates to obtain pure single colonies. Pure single colonies were cryopreserved in TSB with 15% glycerol at -80°C for subsequent DNA extraction.

### Genomic DNA extraction

Bacterial genomic DNA was extracted from pools of sterilized *Ae. aegypti* larvae and bacterial cells retained on the other half of each filter membrane using the Master Pure Gram Positive DNA purification kit® (Epicentre Biotechnologies, Madison, USA) following manufacturer’s instructions. Each sample was disrupted in 150 μl Tris-EDTA (TE) buffer solution with the aid of sterile plastic pestles before DNA extraction.

DNA from pure bacterial colonies was extracted by boiling. Cryopreserved pure bacterial colonies were regrown on TSA at 37 ± 0.5°C for 24 hours prior to DNA extraction. Three to six colonies, depending on size, were picked from TSA plates and mixed with 100 μl of DNA free water in sterile 1.7 ml Eppendorf tubes. The cell suspensions were held in a boiling water-bath for 10 min to lyse the cells, then vigorously homogenized with a vortex mixer for 10 sec and chilled on ice. Resulting DNA samples were stored in -20°C for PCR.

### PCR amplification of bacterial small subunit rRNA gene and TTGE analysis

Bacterial DNA from mosquito larvae and water samples were used as PCR templates. For mosquito and uncultured water samples, the V2-V3 region of the 16S rRNA gene was amplified using universal 16S rRNA bacterial primers HDA1: 5′-ACTC CTA CGG GAG GCA GCA GT-3′, and HDA2: 5′-GTATTA CCG CGG CTG CTG GCA-3′, which yield PCR fragments of ~199 bp. The 16S rRNA gene from cultured water samples was amplified using the primers 27f: 5′-GTGCTGCAGAGAGTTTGATCCTGGCTCAG-3′), and 1492r: 5′-CACGGATCCTACGGGTACCTTGTTACGACTT-3′, which yield PCR fragments of ~1469 bp. Amplification was carried out following previously reported protocols [21] and [18] respectively. PCR products obtained from mosquito larvae and uncultured water samples were separated by TTGE. TTGE migration was performed following a previously reported protocol [21] on 16 cm × 16 cm × 1 mm gels using the DCode universal mutation detection system (Bio-Rad Laboratories, Marne-la-Coquette, France). PCR products from pure bacterial cultures and about 50% of the bands produced by TTGE were sequenced. The remaining bands were assigned to an Operational Taxonomic Unit (OTU) by comparing their migration distance to that of sequenced bands.

### Sequence analysis, taxonomy assignment and alignment

The sequences were analyzed using the Quantitative Insights Into Microbial Ecology (QIIME) software package version 1.7.0 [22]. Bacterial diversity of a sample analyzed using TTGE is generally reflected by the number of bands on the TTGE profile, each band usually representing an OTU. However, Manguin *et al*. [21] reported bands with different migration distances belonging to the same OTU due to sequence heterogeneity among their 16S rRNA gene copies. This could lead to an overestimation of the bacterial diversity. Therefore to avoid this, sequences obtained from TTGE as well as those obtained from water sample cultures were clustered into OTUs, prior to analysis. Sequences were clustered into OTUs using the UCLUST pipeline [23] in QIIME at an identity threshold of 97% (i.e. sequences that were 97% similar were binned into the same OTU). The most abundant sequence within each cluster was selected as a representative of the OTU. The resulting OTUs were assigned to taxa using the Ribosomal Database Project (RDP-II) classifier [24] trained on Greengenes reference database [25] via QIIME at a minimum confidence score of 80%. The OTUs were aligned against the Greengenes core reference alignment [26] using the Python Nearest Alignment Space Termination (PyNAST) alignment algorithm [27] in QIIME with a minimum identity of 75%. Relative abundance of bacterial taxa was computed and collated using the make_OTU_table.py and summarize_taxa.py scripts in QIIME. This was visualized on histograms and compared using two-sample t-tests in Excel. A phylogenetic tree, required to run diversity analyses down the QIIME pipeline, was constructed using the FastTree approximately maximum likelihood program [28] in QIIME. Representative OTU sequences generated from this study are available in the GenBank database [GenBank:KJ814977 - KJ815004; KM108474 - KM108577].

### Diversity analysis

Diversity analysis was performed separately on bacterial sequences obtained by TTGE (mosquito and uncultured water samples), and on those obtained from water samples cultures. The OTU diversity within and between samples were compared, using alpha (α) and beta (β) diversity indices respectively. Alpha diversity was measured with the Shannon-Wiener index (relative OTU abundance and evenness), Observed species metrics (OTU abundance), and Faith’s whole tree Phylogenetic Diversity (branch length-based diversity) [29]. Alpha diversity means and two-sample t-tests were computed using Excel. Beta diversity was evaluated using the unweighted UniFrac [30] pipeline in QIIME. Principal Coordinate Analysis (PCoA) was used to interpret and visualize the variation in UniFrac distance matrix. The largest amount of variation is explained by the first principal coordinate (PCo1), the second largest by the second principal coordinates (PCo2) and so on. To test the strength and significance of the PCoA, the adonis function in PRIMER 6 (PRIMER-E Ltd., Luton, UK) was used on unweighted UniFrac distance matrices via QIIME. The adonis function computes an effect size value R^2^, which shows the percentage of variation in distance matrices explained by the sample. Two-sample student’s t-test to compare mean unweighted UniFrac distances between samples was calculated using the dissimilarity_mtx_stats.py script in QIIME with results presented in boxplots. Prior to diversity analyses samples were rarefied down to the mean number of sequences per sample – four for samples analyzed by TTGE, and two for water sample cultures – in ten iterations to standardize number of sequence per sample. Level of statistical significance for all analyses was set at p ≤ 0.05.

## Results

### Data summary

TTGE profiles were obtained from 18 (72%) pooled mosquito samples, 22 (88%) mosquito positive and 16 (80%) mosquito negative water samples. The remaining samples showed no PCR amplification or faint PCR signals leading to undetectable TTGE profiles. A total of 236 sequences were generated from these TTGE bands; 61 from mosquito larvae, 117 and 58 from mosquito positive and negative water samples respectively. The sequences were binned into 134 unique OTUs, with 1-14 sequences per OTU, and average sequence length of 120 bp. Seven of the unique OTUs failed to align to any bacterial small subunit models (SSU) and were discarded. Sequences from 12 pools of mosquitos, 11 mosquito positive containers, and 10 mosquito negative containers were included in diversity analyses after rarefaction.

Water samples from all mosquito negative containers (17) and 23 (92%) mosquito positive containers produced bacterial colonies on selective media for enteric bacteria. A total of 111 bacterial sequences were obtained from these colonies; 69 from mosquito positive containers and 42 from mosquito negative containers. These sequences were binned into 24 unique OTUs, with 1-5 sequences per OTU, and average sequence length of 911 bp. Seven of the OTUs failed to align to any bacterial SSU models and were discarded. Sequences from 19 mosquito positive, and 13 mosquito negative water samples were included in diversity analyses after rarefaction.

### Alpha diversity of bacterial OTUs

**Figure 1:**
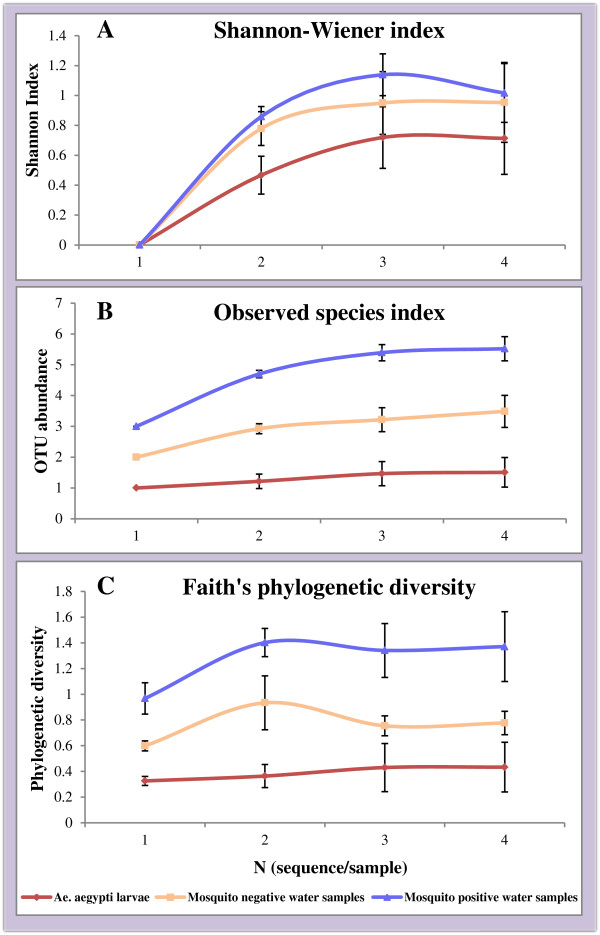
**Alpha diversity rarefaction curves of bacterial OTUs from TTGE.** Alpha diversity rarefaction curves of bacterial OTUs from *Ae. aegypti* larvae, mosquito positive and mosquito negative water samples analyzed by TTGE. This is based on Shannon-Wiener index **(A)**, Observed species index **(B)** and Faith’s phylogenetic diversity **(C)**. Curves represent mean diversity indices for each sample, and error bars represent standard error of means.

Figure 1 shows alpha diversity rarefaction curves based on the Shannon Wiener index, observed species index and Faith’s phylogenetic diversity for sequences generated by TTGE. Mosquito positive water samples had the highest OTU abundance, while mosquito larvae had the lowest across all the three indices (Figure 1). Two-sample t-tests comparing each index between samples revealed statistically significant results for only the Shannon-Wiener index, which was significantly higher in mosquito positive water samples compared to mosquito larvae (p < 0.05). The difference was not statistically significant between mosquito larvae and mosquito negative water samples, or between mosquito positive and negative water samples.

For sequences obtained from bacterial cultures of water samples, OTU abundance was higher in mosquito positive containers compared to mosquito negative containers across all three indices, but the difference was not statistically significant (p > 0.05).

### Beta diversity

**Figure 2:**
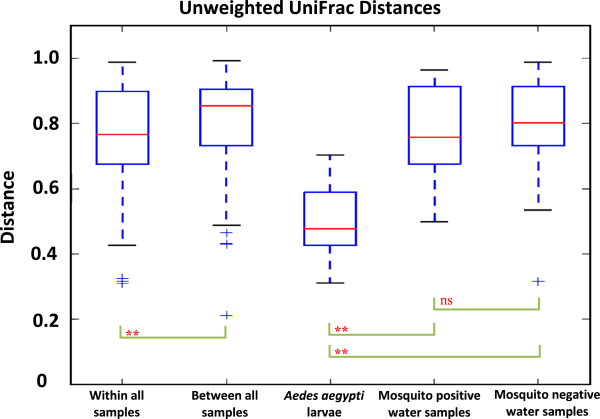
**Boxplots of unweighted UniFrac distances of bacterial OTUs from TTGE.** Boxplots show distribution of unweighted Unifrac distances of bacterial operational taxonomic units (OTUs) within and between *Ae. aegypti* larvae, mosquito positive and mosquito negative water samples. Brackets show outcomes of two-sample t-test comparisons of unweighted UniFrac distances; **P < 0.01; ns, not significant.

**Figure 3:**
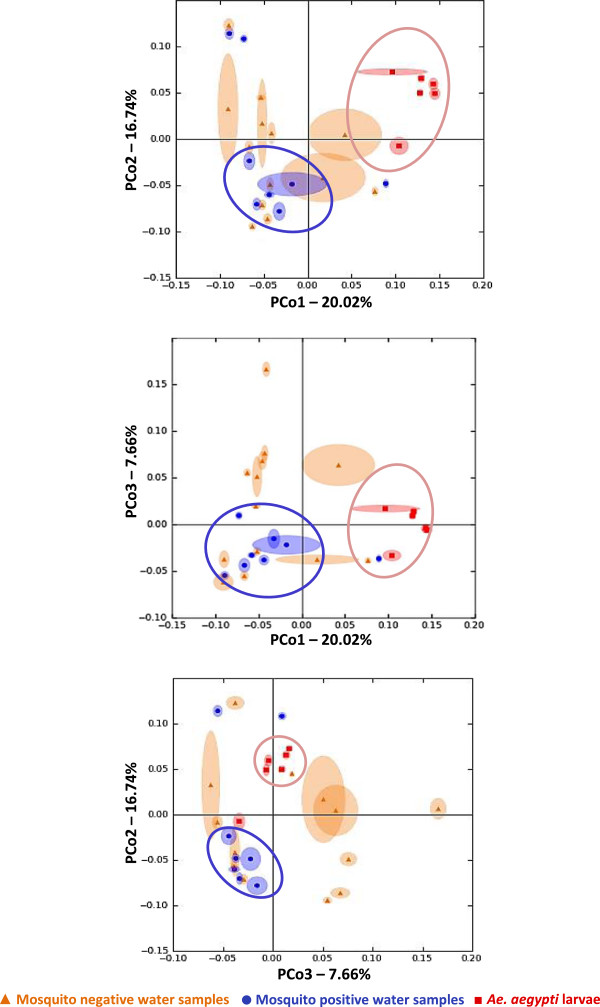
**PCoA of unweighted UniFrac distances between samples analyzed by TTGE.** Principal coordinate analysis of unweighted UniFrac distances between *Ae. aegypti* larvae and water from mosquito positive and negative containers. Distinct clusters of larval samples (red) and mosquito positive water samples (blue) are captured on all three PCoA plots. Mosquito negative water samples (orange) do not show any distinct clusters on any of the plots. Each axis show percentage of variation explained. Each data point consists of a central point surrounded by ellipsoids that indicate variation in UniFrac distances from rarefaction. This demonstrates that the clustering pattern holds up to subsampling.

The unweighted UniFrac distance metrics was used to compare OTU diversity between samples. The TTGE results showed significantly different and lower bacterial diversity in *Ae. aegypti* larvae compared to mosquito positive (p < 0.0001) and negative (p < 0.0001) water samples (Figure 2). There was no significant difference in bacterial diversity between mosquito positive and negative water samples (p > 0.05). The PCoA captured over 44% of variation in UniFrac distances, and revealed distinct clustering patterns of *Ae. aegypti* larvae and mosquito positive water samples (Figure 3). The PCoA plot shows a distinct cluster of *Ae. aegypti* larvae towards the right of the first plot between PCo1 and PCo2, as well as the second plot between PCo1 and PCo3. This cluster is also visible towards the left of the third plot between PCo2 and PCo3 (Figure 3). On all three PCoA plots, mosquito positive water samples cluster towards the bottom left, separately from mosquito larvae (Figure 3). The mosquito negative water samples seem to spread out over all three plots without any distinct clustering (Figure 3). The adonis test, used to test the strength and significance of sample clustering, showed that 20% of the variation explained by PCoA was statistically significant (R^2^ = 0.20, p < 0.001). This indicates that the largest amount of variation, explained by PCo1 (Figure 3), is statistically significant.

Comparing sequences from cultured water samples, there was also no significant difference in OTU diversity between mosquito positive and mosquito negative samples (p > 0.05).

### Bacterial taxa associated with TTGE sequences

**Figure 4:**
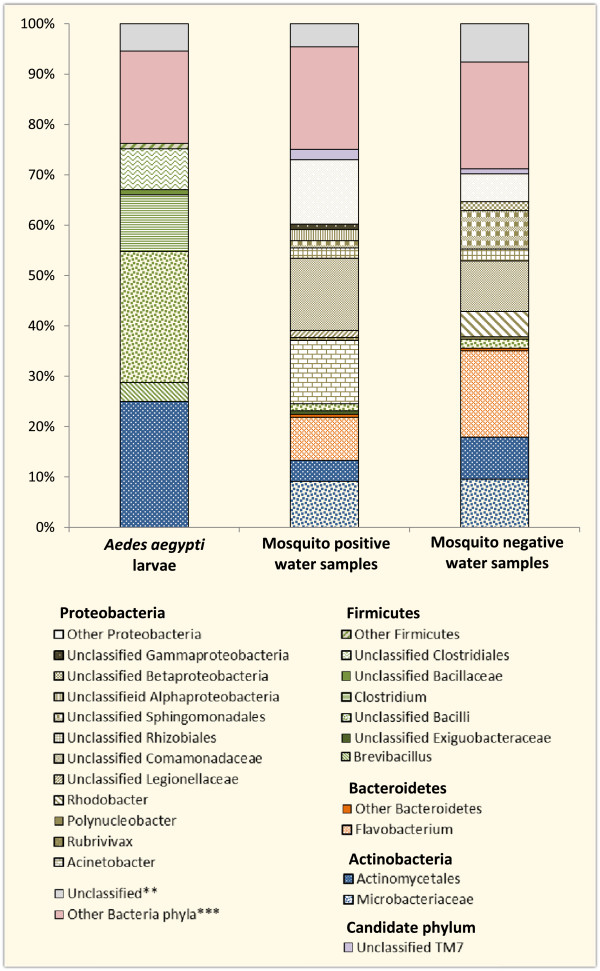
**Relative abundance of bacterial taxa obtained from TTGE sequences.** Bars show mean relative abundance of the different bacterial taxa isolated by TTGE from *Ae. aegypti* larvae, mosquito positive and mosquito negative water samples. **Sequences that were not classified by Qiime. ***Low abundance phyla that are automatically grouped together by QIIME.

Four bacterial phyla and one candidate phylum (TM7) - a major lineage of bacteria for which no cultured representatives have been found, but whose existence is known from environmental 16S rRNA sequences - were identified from *Ae. aegypti* larvae and water from domestic water containers (Figure 4). Only two of these phyla, Actinobacteria and Firmicutes, were found across all samples. The most abundant phylum identified from *Ae. aegypti* samples, was Firmicutes (52%), followed by Actinobacteria (25%). Other bacteria phyla (not broken down in QIIME) made up 18% of the mosquito samples and the remaining 5% were unclassified sequences. Proteobacteria was the predominant phylum in both mosquito positive and negative water samples. It constituted 50% of bacteria phyla found in mosquito positive water samples, followed by Actinobacteria (13%), Bacteroidetes (9%), Firmicutes (2%), TM7 (2%), other phyla (20%), and unclassified sequences (4%). In the mosquito negative water samples, Proteobacteria made up 33% of the total bacteria phyla, followed by Actinobacteria (18%), Bacteroidetes (18%), Firmicutes (2%), TM7 (1%), other phyla (21%) and unclassified sequences (7%).

**Figure 5:**
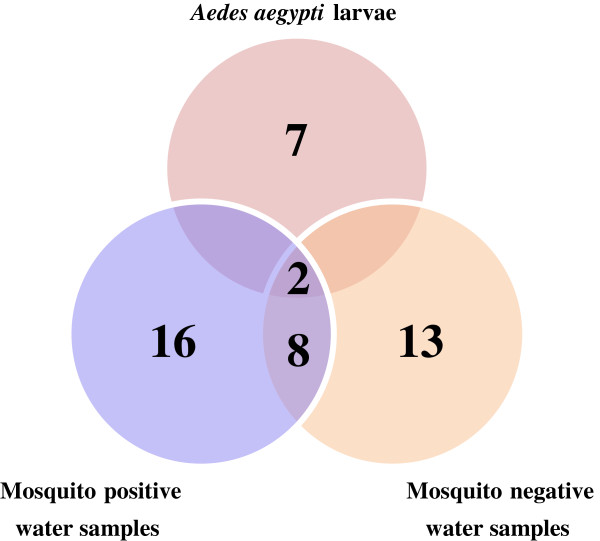
**Number of bacterial taxa obtained from TTGE sequences.** Venn diagram showing number of bacterial taxa obtained from TTGE analysis of *Ae. aegypti* larvae, mosquito positive and mosquito negative water samples.

Figure 5 shows the number of bacterial taxa obtained from TTGE sequences. A total of 24 taxa were identified overall, seven were identified in larval samples, 16 in mosquito positive and 13 in mosquito negative water samples (Figures 4 and 5). Eight of these taxa were shared between mosquito positive and negative water samples (Figure 5), while only two taxa, unclassified Bacilli and Actinomycetales, were common to *Ae. aegypti* larvae and both mosquito positive and negative water samples (Figures 4 and 5). Other than the two bacterial taxa common to all three samples, no other taxon was shared between *Ae. aegypti* larvae and water from either mosquito positive or negative containers (Figure 5). Unclassified Bacilli (26%) were the most abundant bacterial taxa in *Ae. aegypti* larvae, closely followed by unclassified Actinomycetales (25%) and *Clostridium* (11%). The remaining 38% comprised unclassified Clostridiales (8%), *Brevibacillus* (4%), unclassified Bacillaceae (1%), unclassified Firmicutes (1%), other bacterial genera (19%) and unclassified sequences (5%). Unclassified *Comamonadaceae* (15%) were the most abundant in mosquito positive water samples, followed by *Acinetobacter* (13%), unclassified Proteobacteria (13%), unclassified *Microbacteraceae* (9%), *Flavobacterium* (9%), and unclassified Actinomycetales (4%). The remaining 37% comprised other bacterial genera (20%), unclassified sequences (4%) and other small bacterial taxa contributing 1-2% of total bacteria genera (Figure 4). Similar groups of bacteria dominated mosquito negative water samples, with *Flavobacterium* (17%) being the most dominant, followed by unclassified *Comamonadaceae* (10%), unclassified *Microbacteraceae* (10%), unclassified Actinomycetales (8%), unclassified Sphingomonadales (8%), unclassified Proteobacteria (6%), and *Rhodobacter* (5%). The remaining 36% comprised other bacterial taxa (21%), unclassified sequences (7%), and small bacterial taxa contributing 1-2% of total bacterial taxa (Figure 4).

### Bacterial taxa associated with sequences from cultured water samples

**Figure 6:**
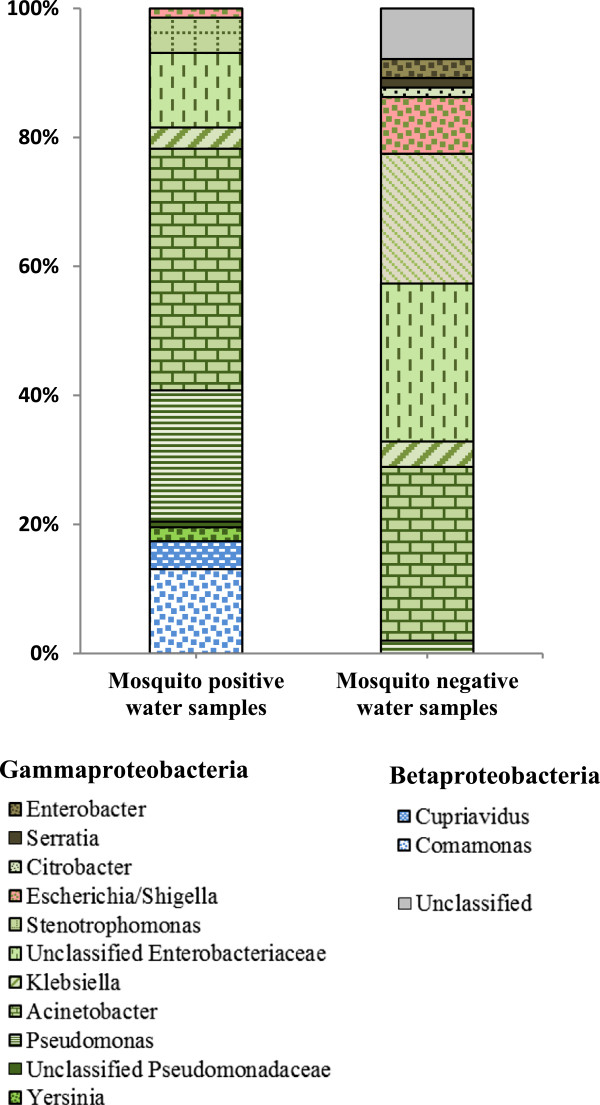
**Relative abundance of bacterial taxa obtained from cultured water samples.** Bars represent mean relative abundance of bacterial taxa isolated from mosquito positive and negative water samples cultured on drigalski agar.

Thirteen bacterial taxa belonging to the phylum Proteobacteria were identified from both mosquito positive and negative water samples (Figure 6). Bacteria belonging to the class Gammaproteobacteria made up 85% (n = 11) of the identified taxa. The other 15% (n = 2) comprised members of the class Betaproteobacteria and were only isolated from mosquito positive water samples (Figure 6). Four of the identified bacterial taxa were exclusively from mosquito positive water samples, three from mosquito negative water samples, and six were common to both. *Acinetobacter* was the most common bacterial genera isolated from mosquito positive water samples. It constituted 37% of the identified bacterial taxa, followed by *Pseudomonas* (20%), *Comamonas* (13%), and unclassified Enterobacteriaceae (12%). The remaining 18% comprised small bacterial taxa, including *Escherichia/Shigella*, making up between 0.8 – 5% of identified bacterial taxa (Figure 6). *Acinetobacter* also dominated mosquito negative water samples, making up 27% of identified bacterial taxa. This was followed by unclassified Enterobacteriaceae (25%), *Stenotrophomonas* (20%) and *Escherichia/Shigella* (9%). The remaining 19% was made up of unclassified sequences (7%), and small bacterial genera contributing 1-4% of identified bacterial taxa.

## Discussion

Very sparse information is available on the nature of the microbial community associated with *Ae. aegypti* larvae in domestic water containers in and around human dwellings. Following our previous study [19], we hypothesized that the bacteria in *Ae. aegypti* infested containers are determinants of *Ae. aegypti* production and thus may constitute a major proportion of the larval microbiota. To test this, we utilized the 16S rRNA-TTGE to comparatively assess the bacterial communities associated with *Ae. aegypti* larvae and water from these containers. In addition to TTGE, water samples were cultured for enteric bacteria. This, to the best of our knowledge, is the first study to compare bacterial communities associated with *Ae. aegypti* larvae and water from domestic containers.

*Aedes aegypti* larvae showed significantly lower OTU abundance (Figures 1 and 2) compared to water samples from domestic containers. This low abundance of bacterial taxa in mosquitoes compared to their breeding habitats has been frequently reported [5, 31, 32], indicating that mosquito larvae naturally have a low number of bacterial taxa. It may also mean that the majority of the bacterial taxa within mosquito larvae are yet to be described, or that methods used for screening are not sufficient to obtain the whole picture of larval microbiota. While larvae consume bacteria, other microbes such as algae and fungi may contribute more significantly to their diet [33, 34], which could be another reason for low bacterial OTU abundance.

Although the impact of mosquito larvae on bacterial abundance and diversity in laboratory and field microcosms have been variable [35], most studies report that the presence of larvae affects the bacterial diversity in breeding habitats [36–38]. Our study showed significantly different bacterial diversity between larvae and water samples (Figures 2, 3, and 4), with little overlap between the bacterial communities. This may be because bacteria already present in the larvae dominate and control the larval microbiota, thereby inhibiting establishment of new bacteria. Factors such as developmental stage, tissue tropism, genetics, dynamics of intra- and inter-specific interactions, as well as environmental factors are thought to influence the bacterial diversity within mosquitoes [10]. In humans and other mammals, gut microbiota play an important role in ‘colony resistance’ , where they prevent colonization by other bacteria or pathogens [9]. There is some evidence of this ‘colony resistance’ in insects [9, 10], as mosquito gut bacteria can either support or suppress the growth of other species by producing inhibitory factors [39]. Bacteria free in the mosquito gut lumen might evoke a host immune-defense response, or modify the gut environment to inhibit development of other bacteria [10, 40]. It is also possible that bacteria taken up by late larval instars from their breeding containers are unable to colonize the mosquitoes at this stage of larval development. This is possible due to the selective, competitive or protective mechanisms elicited by established bacteria that may have been acquired at early stages of development. Internal competition among bacterial species could also be an explanation, as predominant bacterial taxa in mosquitoes may have some competitive advantage over other taxa [41].

Overall, four bacterial phyla and one candidate phylum (TM7) were identified from *Ae. aegypti* larvae and water samples (Figure 4). Only two of these phyla, Firmicutes and Actinobacteria, were associated with both *Ae. aegypti* larvae and water from their breeding containers, suggesting possible dominance and control of larval bacterial flora by these two phyla. The majority of the bacteria isolated from *Ae. aegypti* larvae belonged to the classes Bacilli (*Bacillaceae, Brevibacillus* & unclassified bacilli), Actinobacteria (*Microbacteriaceae* & unclassified Actinomycetales) and Clostridia (*Clostridium* & unclassified Clostridiales), with Bacilli being the most predominant. This is consistent with findings from other studies where bacteria belonging to these classes have been identified in different mosquito species [15, 42–45] including *Ae. aegypti* [41, 46–50]. One study on the midgut microbiota of *Ae. aegypti* larvae collected from natural breeding habitats in Thailand identified Bacilli (*Bacillus cereus*) as the most predominant bacterial class [48]. In contrast, Gammaproteobacteria was the most abundant class (64%) in *Ae. aegypti* larvae collected from domestic containers in Pune and Ahemedabad, India [9]. The TTGE results in this study did not identify bacteria from this class or phylum in *Ae. aegypti* larvae. This may be due to differences in location or techniques used for isolating bacteria, or both.

Proteobacteria was the most predominant phylum in both mosquito positive and negative water samples. The TTGE results showed that Gammaproteobacteria (*Acinetobacter,* unclassified Gammaproteobacteria, and unclassified Legionellaceae), Betaproteobacteria (*Comamonadaceae, Polynucleobacter, Rubirivivax*, and unclassified betaproteobacteria) and Actinobacteria (Actinomycetales, and Microbacteriaceae), were the most abundant bacterial classes in mosquito positive water samples, with Gammaproteobacteria dominating. Flavobacteria (*Flavobacterium*), Alphaproteobacteria (*Rhodobacter*, unclassified Sphingomonadales, and unclassified Rhizobiales), and Betaproteobacteria (unclassified Comaonadaceae, unclassified Betaproteobacteri and *Polynucleobacter*) were the most abundant in mosquito negative water samples, with Flavobacteria dominating. Enteric bacteria belonging to the class Gammaproteobacteria were isolated from both mosquito positive and negative water samples by selective bacterial culture (Figure 6). This class constituted 15% of the total bacterial population isolated from mosquito positive water samples using TTGE, but was not identified in mosquito negative samples (Figure 4). It may have constituted the ‘other bacterial phyla’ not broken down in QIIME due to negligible proportions. This indicates that the concentration of Gammaproteobacteria may have been below TTGE detection limit, which is still debatable [18], or outweighed by high concentrations of other competing bacterial DNA [51].

Some bacteria are known to pass through 0.45 μm pore size membrane filters impacting bacterial density and, to a lesser extent, diversity. Hence some bacteria in this study may have been lost during filtration. Nonetheless, our results are in line with those of similar studies conducted previously. Studies on the bacterial composition of water samples from domestic containers have understandably focused on fecal contamination [19, 52–54], providing little information on general bacterial composition. In the one study conducted so far, majority of the bacteria isolated from *Ae. aegypti* infested domestic containers were Proteobacteria, predominantly Gammaproteobacteria [9] which is in line with results reported here. In other mosquito habitats such as tree holes, tyres, discarded containers, plant pots, and laboratory mesocosms, Proteobacteria have also been shown to be predominant [5, 9, 44]. Isolation of enteric bacteria from *Ae. aegypti* infested domestic water containers supports findings from our earlier study [19] where these containers were more likely to be contaminated with *E. coli* (an enteric bacteria) than not. This suggests that enteric bacteria may play a role in *Ae. aegypti* infestation in this setting.

## Conclusions

We present for the first time results of the bacterial composition of fourth-instar *Ae. aegypti* larvae and water from *Ae. aegypti* infested domestic water containers. *Aedes aegypti* had significantly lower OTU abundance compared to mosquito positive water samples. There was no significant difference in OTU abundance between larvae and mosquito negative water samples or between mosquito positive and negative water samples. Larval samples had significantly different OTU diversity compared to mosquito positive and negative water samples, with no significant difference between mosquito positive and negative water samples. The TTGE analysis identified a total of 24 bacterial taxa belonging to the phyla Proteobacteria, Firmicutes, Actinobacteria, Bacteroidetes and TM7 (candidate phylum). Seven of these taxa were identified in *Ae. aegypti* larvae, 16 in mosquito positive and 13 in mosquito negative water samples. Eight of the bacterial taxa were common to both mosquito positive and negative water samples, while only two, Firmicutes and Actinobacteria, were common to both larvae and water samples. The lower bacterial abundance in late instar larvae compared to water samples could be due to dominance and control of larval flora by bacteria that may have been established during earlier stages of development. Further investigation of the mosquito-bacteria interactions at all larval stages is needed to understand the dynamics involved. Enteric bacteria were sparsely represented by TTGE, but were isolated from both mosquito positive and negative water samples by selective culture. Isolation of enteric bacteria from water samples in this study supports our previous results of *E. coli* contamination in *Ae. aegypti* infested domestic containers in this setting. Studies are needed to understand the role of enteric bacteria in *Ae. aegypti* infestation of these containers. We also show that TTGE alone may not sufficiently describe the bacterial communities in mosquito and water samples. Supplementing with culture-dependent methods presents a better picture of the bacterial communities.

## Abbreviations

DA: Drigalski agar
DGGE: Denaturing gradient gel electrophoresis
OTU: Operational taxonomic units
PCoA: Principal coordinates analysis
PyNAST: Python nearest alignment space termination
QIIME: Quantitative insights into microbial ecology
RDP: Ribosomal database project
SSU: Small subunit
Tris-EDTA (TE): Ethylenediaminetetraacetic acid-Tris(hydroxymethyl)aminomethane
TSA: Trypticase soy agar
TSB: Tryptic soy broth
TTGE: Temporal temperature gradient gel electrophoresis
UPGMA: Unweighted pair group method with arithmetic mean.
